# An approach to electroanatomical mapping with a pentaspline pulsed field catheter to guide atrial fibrillation ablation

**DOI:** 10.1007/s10840-025-01980-6

**Published:** 2025-03-04

**Authors:** Mark T. Mills, Peter Calvert, Calum Phenton, Nicole Worthington, Derick Todd, Simon Modi, Reza Ashrafi, Richard Snowdon, Dhiraj Gupta, Vishal Luther

**Affiliations:** 1 Liverpool Centre for Cardiovascular Science at University of Liverpool, Liverpool John Moores University and Liverpool Heart & Chest Hospital, Liverpool, UK; 2 Department of Cardiology, Liverpool Heart & Chest Hospital NHS Foundation Trust, Thomas Drive, Liverpool, L14 3PE UK; 3 Abbott Medical UK Ltd, Solihull, UK

## Abstract

**Background:**

Pulsed field ablation (PFA) of atrial fibrillation (AF) using a pentaspline multi-electrode catheter is commonly performed under fluoroscopic guidance. No data exist on the integration of this catheter within a three-dimensional electroanatomical mapping (3D-EAM) system for left atrial voltage and activation mapping, posterior wall isolation (PWI), or redo ablation. This technical report reviews an approach whereby mapping is performed using the pentaspline PFA catheter itself within an open architectural impedance–based 3D-EAM system.

**Methods:**

Cases involved mapping with the PFA catheter itself, with real-time visualisation of the guidewire tip and catheter within the 3D-EAM system. In certain cases, additional 3D-EAM was performed with a grid-style high-density mapping catheter for comparison.

**Results:**

In a series of 22 patients (45% female, mean age 63 ± 13 years, 55% paroxysmal AF, 27% redo procedures), mapping increased procedural times (mean 108 min vs. 68 min in fluoroscopy-only controls), without reducing fluoroscopy times. Three potential advantages of mapping with the PFA catheter were identified: (1) The technique helped identify sleeves of incomplete pulmonary vein isolation after index applications. (2) In the four cases mapped with both the PFA and grid-style catheters, voltage maps appeared concordant. (3) The technique helped facilitate robust PWI and identify inadvertent partial PWI.

**Conclusions:**

3D-EAM with a pentaspline PFA catheter itself is feasible, without the need for high-density mapping catheters. This approach has potential advantages over fluoroscopic-only guidance, although its long-term efficacy and cost-effectiveness require formal assessment.

**Graphical Abstract:**

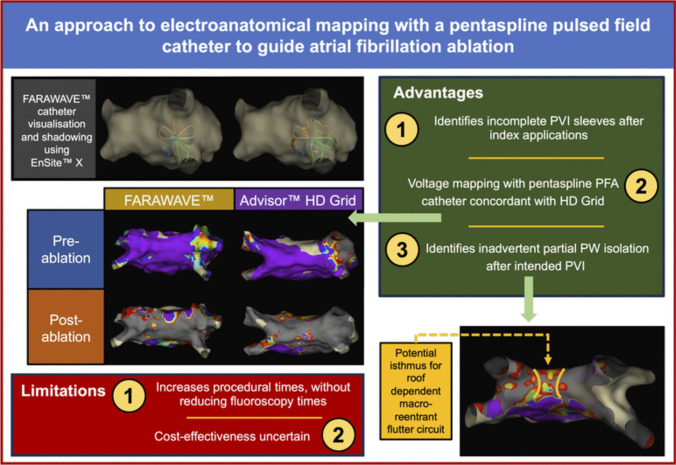

**Supplementary Information:**

The online version contains supplementary material available at 10.1007/s10840-025-01980-6.

## Introduction 

Catheter ablation is an established, guideline-recommended approach to the rhythm control of atrial fibrillation (AF), with pulmonary vein isolation (PVI) forming the cornerstone of therapy [1]. Until recently, AF ablation has been achieved using thermal modalities, namely radiofrequency (RF) and cryoablation [2]. Pulsed field ablation (PFA) is an emerging non-thermal ablation modality, with purported advantages in terms of safety, especially when ablating in proximity to the oesophagus [3, 4]. As a result, left atrial posterior wall (PW) isolation has become particularly attractive during clinical procedures.

The FARAPULSE™ system (Boston Scientific) is one of the first PFA systems to have gained regulatory approval, comprising a single-shot, pentaspline, multi-electrode catheter (FARAWAVE™, Boston Scientific) which is manipulated within the left atrium (LA) over a guidewire, through a dedicated sheath (FARADRIVE™, Boston Scientific). In the majority of cases in Europe, FARAPULSE™ PFA is delivered under fluoroscopic guidance [4–7], enabling visualisation of the guidewire and positioning of the FARAWAVE™ catheter in basket and flower configurations. Our institution is a high-volume FARAPULSE™ PVI centre, recently reporting our experience under fluoroscopic guidance [4]. One-year crude Kaplan–Meier estimates of arrhythmia freedom, after a 2-month blanking period, were 51% (95% confidence interval 42%–60%). Pulmonary vein (PV) reconnections have been reported in studies post fluoroscopic-guided FARAPULSE™ PVI [8–11], highlighting the need for strategies to improve long-term efficacy.

Increasingly, a hybrid of fluoroscopy and three-dimensional electroanatomical mapping (3D-EAM) guidance is utilised for FARAPULSE™ PFA. EnSite™ X (Abbott Medical) is an open architecture 3D-EAM platform with a recent software expansion module that enables visualisation of the FARAWAVE™ catheter (Software Version 3.0.2, PFA Catheter Visualisation license, Abbott Medical). This was recently released for early evaluation in select centres. In this report, we discuss the feasibility of mapping with the FARAWAVE™ catheter itself, summarising potential advantages over fluoroscopic-only guidance, as well as areas where improvements may be necessary.

## Methods

### Study participants

Data from consecutive patients undergoing FARAPULSE™ AF ablation with concomitant EnSite™ X 3D-EAM were prospectively collected during limited market release (between April and June 2024) at our institution (Liverpool Heart and Chest Hospital NHS Foundation Trust, UK).

### Procedural setup

All procedures were performed by electrophysiology consultants who had already gained experience of FARAPULSE™ PFA since mid-2022 under fluoroscopic guidance [4]. Procedures were performed on uninterrupted oral anticoagulation, under general anaesthesia. Transoesophageal echocardiography was undertaken to exclude LA thrombus and guide transeptal puncture. Via femoral venous access, a decapolar catheter was advanced into the coronary sinus (CS). Transeptal puncture was performed using a 63-cm, standard 8-Fr long sheath (Swartz™ braided transseptal guiding introducer Lamp 45™, Abbott Medical) and a BRK-1 needle. After transeptal puncture, heparin was administered, with an activated clotting time target of > 300 s throughout the procedure. The Lamp 45™ sheath was exchanged over an InQwire® guidewire (Merit Medical) for the 13.8-Fr (inner diameter) FARADRIVE™ sheath. A 31-mm FARAWAVE™ ablation catheter was advanced into the LA.

### 3D-Mapping with FARAWAVE™

**Fig. 1:**
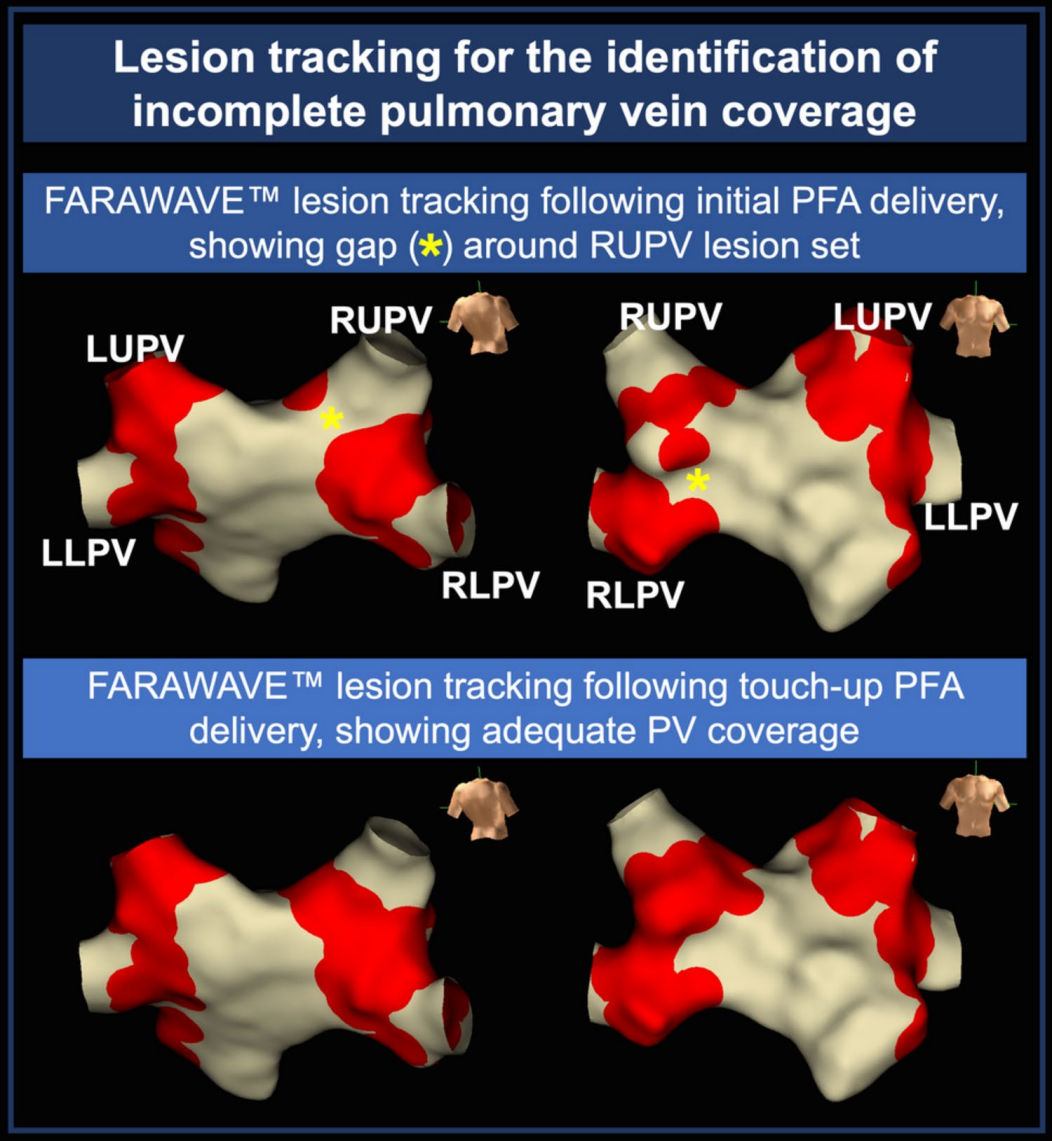
FARAWAVE™ catheter lesion tracking for the identification of incomplete pulmonary vein coverage. Top: Review of FARAWAVE™ lesion tracking (red) after eight applications per pulmonary vein identifies potential gaps (yellow asterisks) in the right upper pulmonary vein lesion set. Bottom: Lesion tracking after a further two FARAWAVE™ applications showing appropriate pulmonary vein coverage. LLPV, left lower pulmonary vein; LUPV, left upper pulmonary vein; PFA, pulsed field ablation; PV, pulmonary vein; RLPV, right lower pulmonary vein; RUPV, right lower pulmonary vein

The FARAWAVE™ catheter was connected to the catheter interface module (CIM) on the EnSite™ X EP system using the NavX™ impedance mapping modality. The FARAWAVE™ catheter comprised five splines, with four electrodes per spline (20 in total). A bipole was derived for each of the five splines, with the distal electrode represented by the interpolation of electrodes 1, 2 and 4 of each spline and electrode 3 as the proximal electrode. As such, only electrode 3 of each spline could record signals and be located on the EnSite™ X system (Supplementary Material – Fig. 1). The five FARAWAVE™ splines were visualised in either flower or basket configuration, with manual toggling between the two configurations under operator guidance to ensure appropriate rendering. The InQwire® guidewire was also connected to the CIM using alligator clips, to create a unipolar electrode, allowing visualisation of the guidewire tip as an orb in the EAM system (Supplementary Material – Video 1).

A 3D-EAM of the LA and PVs was collected using continuous anatomy sampling from the five FARAWAVE™ bipoles during CS pacing at 600 ms. Patients in AF at procedure start were cardioverted to sinus rhythm prior to mapping. The catheter in flower configuration, combined with the flexing ability of the sheath, allowed for rapid and smooth LA map creation along the roof, PW and floor. PV mapping was performed with the catheter in olive/basket configuration. In certain cases, supplementary high-density 3D-EAM was performed using an Advisor™ HD Grid mapping catheter (Abbott Medical); the decision to use the HD Grid was at operator discretion and was generally used in operators’ first cases with the novel mapping software. We performed an offline analysis of left atrial low-voltage area pre- and post-ablation using the FARAWAVE™ and Advisor™ HD Grid catheters, choosing to define low voltage (“scar”) as tissue with a bipolar voltage < 0.3 mV (based on our work [12] and histological validation [13] studies).

### PFA lesion tracking and catheter shadowing

Within the Ensite™ X map, surrogate lesion markers can be visualised at the five FARAWAVE™ electrodes at the time of PFA delivery using an adapted local activation time map. These are represented as red circles on the 3D model and can be collected in either catheter shape configuration. This allows for visual assessment of PV ablation coverage after PFA delivery and is shown in Fig. 1.

**Fig. 2:**
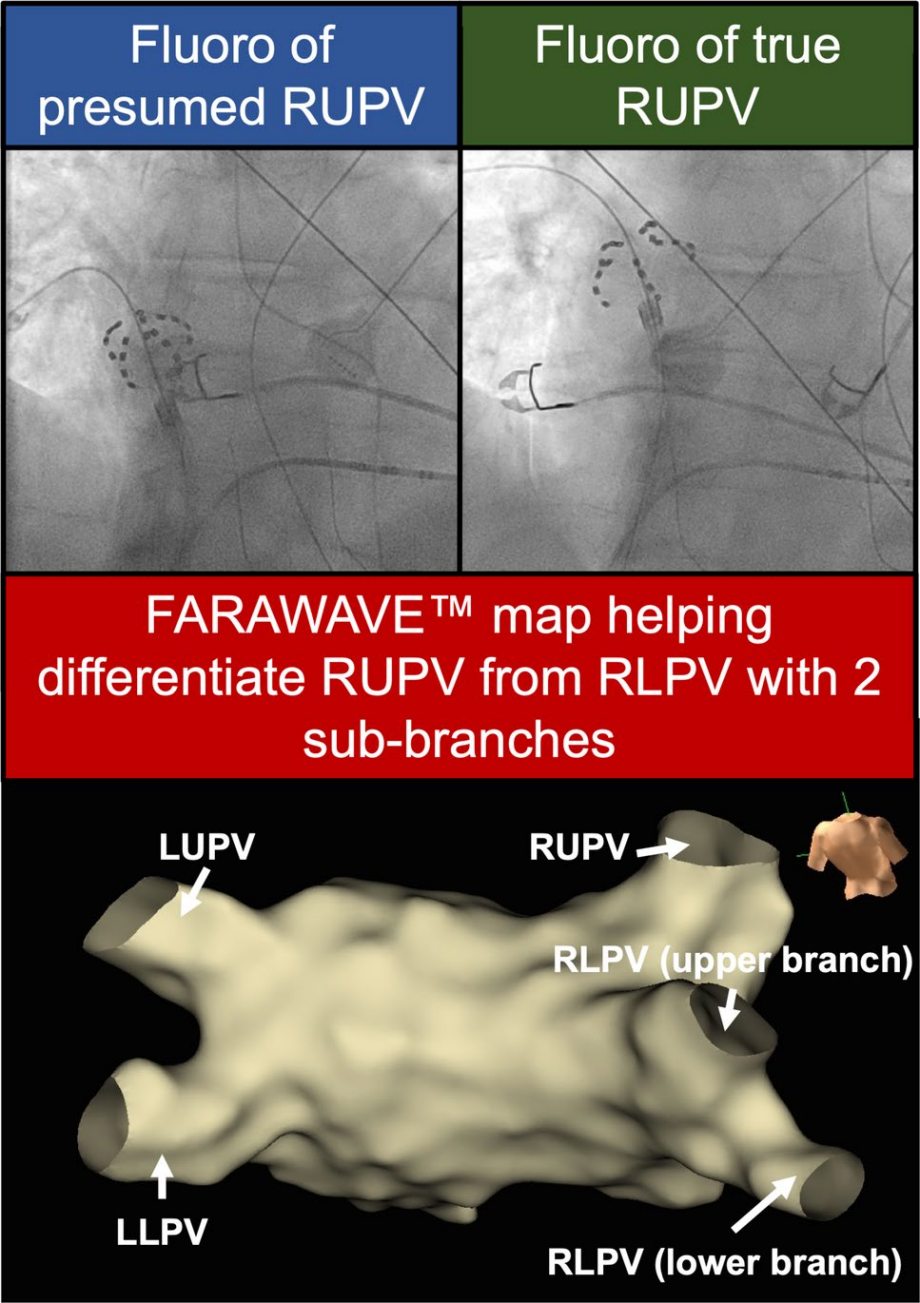
Insights into individual anatomy. Top left: Fluoroscopic image of the FARAWAVE™ catheter in a presumed right upper pulmonary vein. Bottom: Subsequent FARAWAVE™ map (postero-anterior view) demonstrating another vein above the fluoroscopically identified right upper pulmonary vein, with confirmation of a right lower pulmonary vein with two sub-branches, and a true right upper pulmonary vein above this. Top right: The true right upper pulmonary vein was subsequently identified on repeat fluoroscopy. Fluoro, fluoroscopy image; LLPV, left lower pulmonary vein; LUPV, left upper pulmonary vein; RLPV, right lower pulmonary vein; RUPV, right lower pulmonary vein

**Fig. 3:**
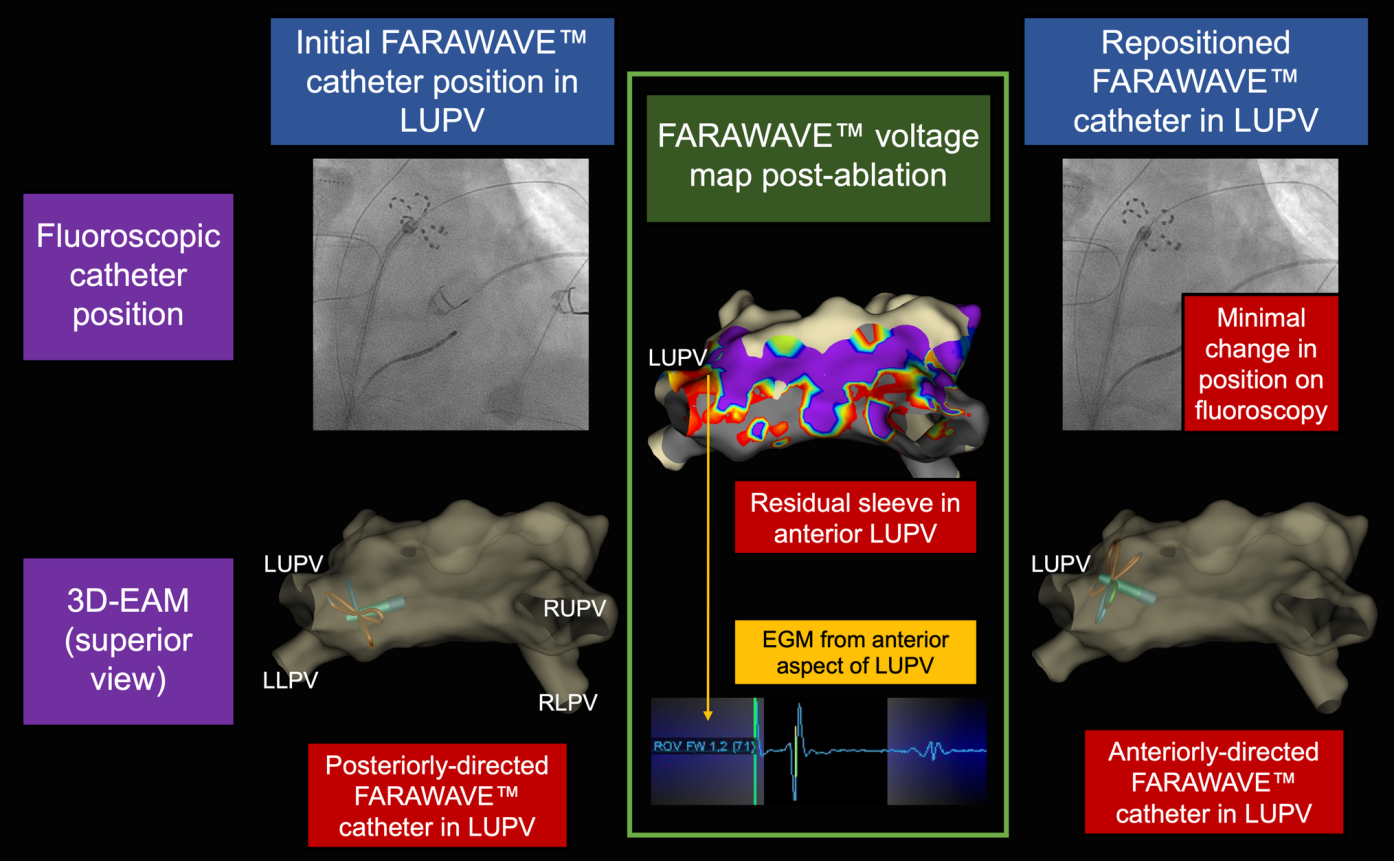
FARAWAVE™ mapping allows for appreciation of anterior/posterior orientation of the ablation catheter in the pulmonary vein over fluoroscopic guidance. Left-hand images: Posteriorly directed FARAWAVE™ catheter in left upper pulmonary vein and concordant position on fluoroscopy (right anterior oblique view). Middle images: FARAWAVE™ voltage map post-ablation demonstrating anterior sleeve in left upper pulmonary vein (and in right upper pulmonary vein), with intracardiac electrogram from the left upper pulmonary vein. Right-hand images: Anteriorly directed FARAWAVE™ catheter in left upper pulmonary vein following catheter repositioning, with concordant fluoroscopic image showing minimal change in position compared to previous fluoroscopy. Orthogonal fluoroscopic views were not available for comparison. EGM, electrogram; LLPV, left lower pulmonary vein; LUPV, left upper pulmonary vein; RLPV, right lower pulmonary vein; RUPV, right lower pulmonary vein

The EnSite™ X mapping system allows for real-time visualisation of catheter movement in basket and flower configurations. By creating a ‘shadow’ of the catheter at the location of PFA delivery, subsequent catheter movements and rotations can be compared to prior applications without fluoroscopic guidance, ensuring appropriate positioning within each PV (Supplementary Material – Fig. 2). For PW isolation (PWI), visualisation of the catheter on the LA PW, with projection of catheter shadows and lesion tracking, was used to guide ablation (Supplementary Material –Fig. 3).

### Ablation

In first-time ablation cases, PFA was delivered with the 31-mm FARAWAVE™ catheter as per manufacturer recommendations, with a minimum of eight energy applications to each PV at 2.0 kW (four in basket and four in flower configuration). In redo cases, prior PVI was assessed at procedure start, with PFA delivered to non-isolated PVs and/or to PV ostia to extend the antral ablation line. In a subset of cases, LA PWI was performed using the FARAWAVE™ catheter under 3D-EAM visualisation, with the catheter in flower configuration and the guidewire retracted into the sheath. A minimum of two applications were delivered at each PW site, ensuring adequate overlap between catheter positions using the ‘shadow’ function. PFA was limited to LA ablation; if cavotricuspid isthmus (CTI) ablation was required, this was conducted using RF ablation. LA RF ablation was not performed.

### Outcomes

Outcomes included acute ablation success (defined as evidence of PVI and/or PWI (if performed) confirmed through intracardiac electrograms and 3D-EAM) and procedural metrics (skin-to-skin procedural time, fluoroscopy time). Qualitative operator feedback was collected to identify perceived advantages and limitations of mapping with the FARAWAVE™ catheter, and a thematic analysis was performed to identify common learning points, presented as ‘lessons’.

### Design and consent

As an observational series over a fixed 2-month period, no formal research protocol was implemented. Concomitant fluoroscopic guidance was permitted. The decision whether to map with the FARAWAVE™ catheter map before or after ablation—and whether to perform additional mapping with the Advisor™ HD Grid catheter at either time point—was at operator discretion. All patients provided informed written consent for their clinical procedure and additional consent for inclusion in this publication.

## Results

Patient demographics and procedural characteristics are summarised in Table 1. Overall, 22 patients were included (45% female, mean age 63 ± 13 years, 55% paroxysmal AF). Six patients had previously undergone AF ablation.

**Table 1:** Patient demographics and procedural characteristics

Patients (*n*)	22
Age (mean ± SD), years	63 ± 13
Female (*n* [%])	10 (45)
Paroxysmal AF (*n* [%])	12 (55)
Left atrial size	
• Normal	7 (32)
• Mildly dilated	5 (23)
• Moderately dilated	4 (18)
• Severely dilated	6 (27)
Previous AF ablation (*n* [%])	6 (27)
Skin-to-skin procedure time (median [IQR]), mins	108 [94–123]
Fluoroscopy time (median [IQR]), mins	20 [12–26]
Extra-PV ablation	
• PWI (*n* [%])	6 (27)
• CTI (*n* [%])	6 (27)

*AF* atrial fibrillation, *CTI* cavotricuspid isthmus, *IQR* interquartile range, *PV* pulmonary vein, *PWI* posterior wall isolation, *SD* standard deviation

Median skin-to-skin procedure time was 108 min (interquartile range [IQR] 94–123 min), whilst median fluoroscopy time was 20 min (12–26 min). PWI was performed in six patients, and RF CTI in three cases. Acute AF ablation success was 100%.

The majority of patients (55%) underwent mapping with the FARAWAVE™ catheter only (pre- and post-ablation). Four patients (18%) underwent mapping with both the FARAWAVE™ and Advisor™ HD Grid catheters pre- and post-ablation, whilst two had mapping with the Advisor™ HD grid pre-ablation but not post-ablation. Other mapping combinations in individual cases are highlighted in Supplementary Material –Table 1. Median mapping time with the FARAWAVE™ catheter was 12 min (IQR 11–16 min) prior to PFA application and 9 min (7–13 min) post-ablation.

Excluding redo cases and those where the Advisor™ HD Grid catheter was used, skin-to-skin procedure time was 101 min (93–113 min) and fluoroscopy time 15 min (12–22 min) across 12 patients.

Below, we summarise and discuss operator feedback as three key lessons from our experience of 3D-EAM with the FARAWAVE™ catheter (Central Illustration).

### Lesson 1: Identification of incomplete pulmonary vein isolation

Lesion tracking at the time of PFA delivery was identified as a useful tool in localising areas requiring further ablation after the routine 8 applications per PV. An illustrative case is presented in Fig. 1, where lesion tracking facilitated identification of gaps in the right PV lesion set after initial PFA application, with further targeted ablation applied.

Operators reported that mapping helped define LA and PV anatomy over fluoroscopy alone. Figure 2 illustrates a case where the operator initially believed to have identified the right upper PV (RUPV) on fluoroscopy. However, on subsequent mapping with the FARAWAVE™ catheter, another vein was rapidly identified above the initially presumed RUPV using the guidewire tip, represented as an orb in the 3D-map. In this case, 3D-EAM clearly demonstrated a right lower PV (RLPV) with two sub-branches.

The assessment of adequate catheter positioning on fluoroscopy was often associated with anterior or posterior tilt on 3D-EAM (Fig. 3). Indeed, minor catheter movements—often not appreciable on fluoroscopy—resulted in marked changes in catheter position on the 3D-EAM (e.g. moving the catheter from the posterior to anterior aspect of a PV).

**Fig. 4:**
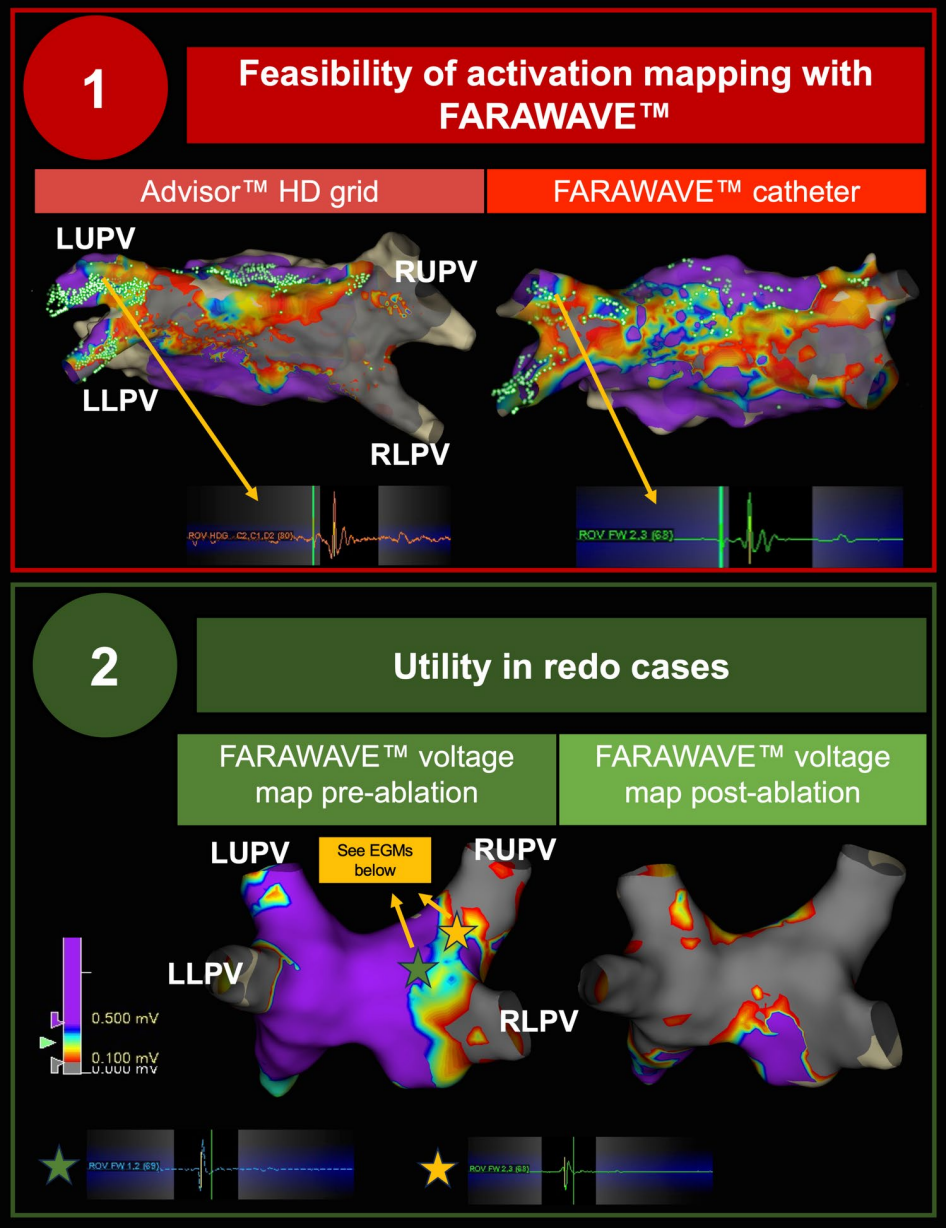
FARAWAVE™ catheter mapping for the identification of incomplete pulmonary vein isolation. (1) Activation mapping is feasible. Sparkle map collected with an Advisor™ HD Grid (left) and a FARAWAVE™ catheter (right) post-pulsed field ablation delivery, demonstrating concordant sleeve of activation into the posterior aspect of the left-sided pulmonary veins, with corresponding electrograms. (2) Utility in redo ablation. Left: FARAWAVE™ voltage map pre-ablation in a patient with long-standing persistent atrial fibrillation and prior radiofrequency pulmonary vein isolation, demonstrating reconnection of all four pulmonary veins. Right: FARAWAVE™ voltage map post-FARAPULSE™ ablation, demonstrating satisfactory pulmonary vein and posterior wall isolation. EGM, electrogram; LLPV, left lower pulmonary vein; LUPV, left upper pulmonary vein; PFA, pulsed field ablation; PV, pulmonary vein; RLPV, right lower pulmonary vein; RUPV, right lower pulmonary vein

In addition, we observed the feasibility of activation mapping with the FARAWAVE™ catheter to identify PV sleeves after ablation. An illustrative case is presented in Fig. 4, showing consistency between post-ablation activation maps collected with the FARAWAVE™ and Advisor™ HD Grid catheters. In this case, both maps demonstrated a gap at the posterior aspect of the left PVs, which was subsequently successfully targeted with additional PFA applications.

In six cases, 3D-EAM was used to guide redo ablation. Figure 4 highlights the case of a patient with persistent AF undergoing redo ablation. A baseline FARAWAVE™ voltage map showed reconnection of all four PVs, which were targeted with PFA applications. Due to AF chronicity, PWI was also performed in this case. A repeat voltage map following PFA delivery confirmed isolation of all four PVs and of the PW.

### Lesson 2: Mapping with the FARAWAVE™ catheter appears concordant with a high-density grid-style catheter

**Fig. 5:**
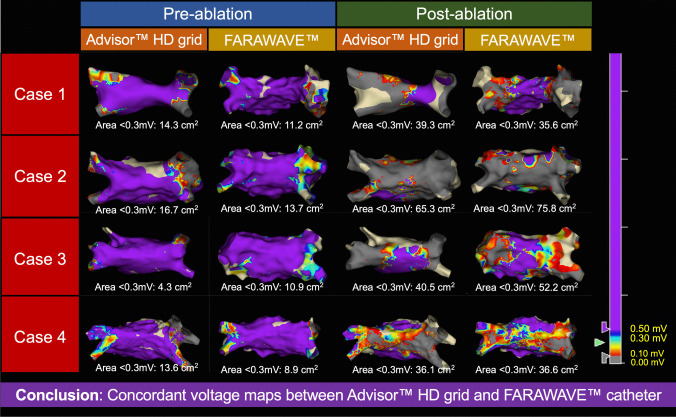
Voltage mapping is feasible with the FARAWAVE™ catheter, producing concordant maps to a high-density mapping catheter. Four cases during which voltage mapping was performed prior to and following pulsed field ablation delivery, with both an Advisor™ HD Grid catheter and the FARAWAVE™ catheter, showing high fidelity between the two techniques (postero-anterior views of the left atrium). Left atrial low voltage area was defined as tissue with a bipolar voltage < 0.3 mV

In our first four cases, we performed detailed left atrial voltage mapping with both an Advisor™ HD Grid and the FARAWAVE™ catheter pre- and post-PFA delivery, in order to compare maps between the two approaches. The four representative voltage maps for each case are summarised in Fig. 5. Pre- and post-ablation maps appeared to show concordance—in terms of both anatomical and voltage data—between the two catheters. Left atrial low voltage area (defined as a bipolar voltage < 0.3 mV) was numerically similar between the two catheters (pre-ablation mean low voltage area 12.3 cm^2^ with Advisor™ HD grid and 11.2 cm^2^ with FARAWAVE™ catheter; post-ablation mean low voltage area 45.3 cm^2^ with Advisor™ HD grid and 50.1 cm^2^ with FARAWAVE™ catheter).

### Lesson 3: Mapping facilitates robust posterior wall isolation

Mapping was helpful in delivering robust PWI in planned cases and in avoiding inadvertent PWI in cases where only PVI was intended. By integrating the FARAWAVE™ catheter into the 3D-EAM, procedural workflow could be modified by visualising the catheter position on the PW, using the ‘shadow’ function to ensure overlap of applications, using the lesion tracking tool to ensure adequate coverage, and by performing post-ablation voltage mapping to confirm successful isolation (Supplementary Material –Fig. 3).

**Fig. 6:**
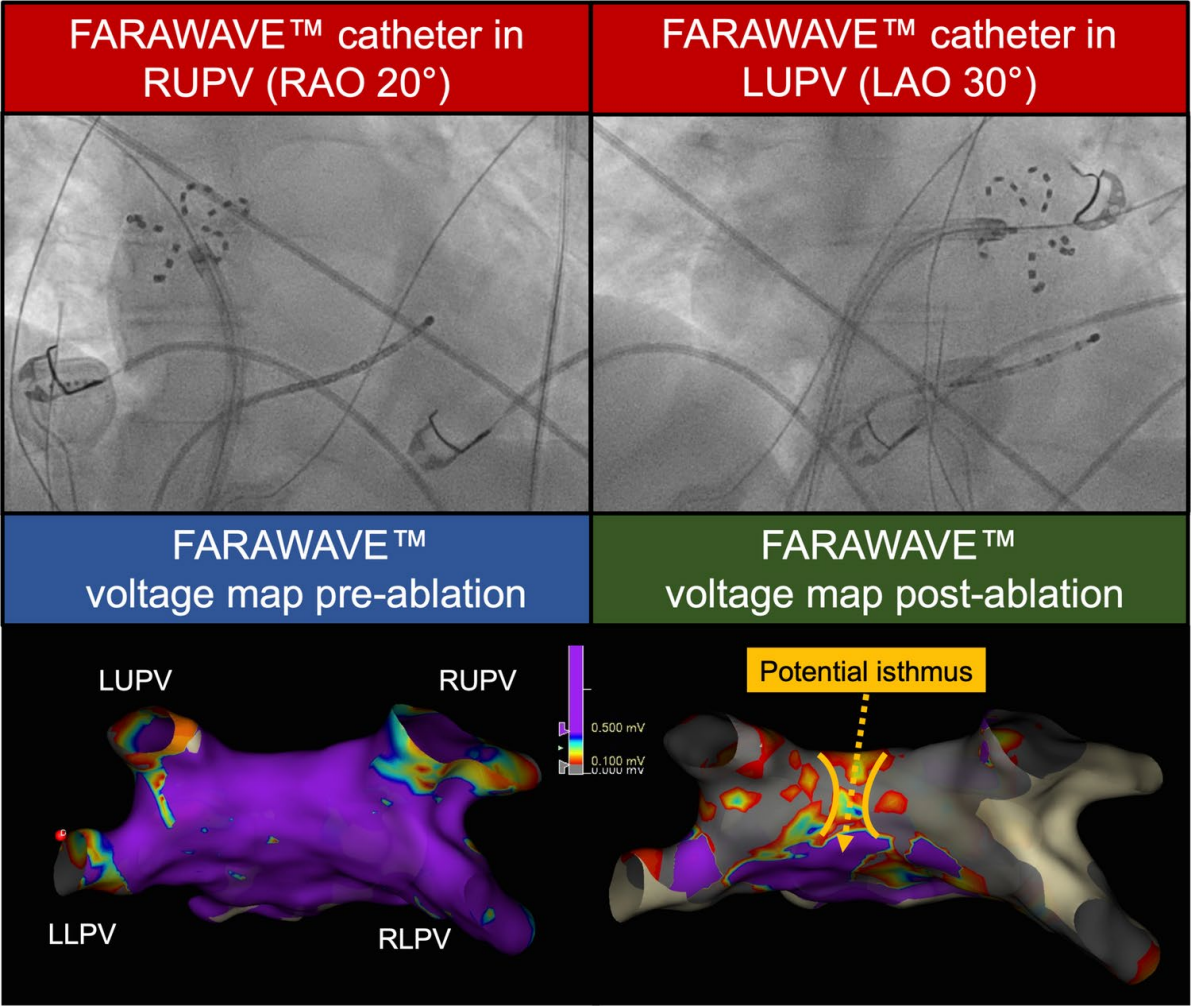
Example of inadvertent partial posterior wall isolation identified through FARAWAVE™ voltage mapping. A patient with paroxysmal atrial fibrillation undergoing FARAPULSE™ pulmonary vein isolation. Top: Fluoroscopic images of the FARAWAVE™ catheter in flower configuration in the right upper pulmonary vein (left-hand image) and left upper pulmonary vein (right-hand image). Bottom left: Baseline FARAWAVE™ voltage map prior to pulsed field ablation delivery demonstrating preserved left atrial voltages. Bottom right: Post-ablation FARAWAVE™ voltage map demonstrating low voltage areas on the posterior wall, with a probable central isthmus

In Fig. 6, we present the case of a patient with a normal sized LA undergoing PVI for paroxysmal AF using a 31-mm FARAWAVE™ catheter. A 3D-EAM was created with the FARAWAVE™ catheter, showing preserved voltages at baseline. Fluoroscopic views of the FARAWAVE™ catheter in the left and right upper PVs demonstrated satisfactory catheter positioning. A FARAWAVE™ voltage map post-ablation demonstrated low voltages across the majority of the PW, with a possible narrow central isthmus of healthier tissue, due to the close proximity and overlap of the left and right-sided PFA applications. This was not fully appreciated at the time of ablation, and further PW ablation to this area was not applied. One week after ablation, the patient presented with new atypical flutter requiring direct current cardioversion; review of the maps highlighted the probable inadvertent—and incomplete—PWI. We hypothesise that further ablation to the PW isthmus during the index procedure may have prevented this.

## Discussion

Our series is the first to report on the feasibility and utility of impedance-based 3D-EAM using the FARAWAVE™ catheter itself for left atrial voltage and activation mapping and guiding redo ablation and PWI. Learning points include the following:

1. The technique may help identify incomplete PVI sleeves after index applications over fluoroscopy alone.
2. Voltage and activation mapping with the pentaspline PFA catheter appeared concordant with the Advisor™ HD Grid catheter.
3. The technique helped facilitate robust PWI and identify inadvertent partial PWI after intended PV applications alone.

Despite initial data from pre-release studies suggesting that FARAPULSE™ PVI may lead to improved AF-free survival compared to thermal ablation [3], subsequent studies have observed disappointingly similar efficacies [4, 14]. Our report offers a plausible hypothesis for this discordance, in that fluoroscopic guidance may lead to sub-optimal PFA delivery, increasing the risk of PV reconnections and creating the substrate for atypical LA flutter circuits through inadvertent left and right PV lesion overlap. Below, we discuss potential advantages and limitations of impedance-based 3D-EAM with the FARAWAVE™ catheter.

### Potential advantages of mapping with the FARAWAVE™ catheter

A detailed understanding of LA anatomy and PV configuration is central to successful ablation. This can be limited with fluoroscopy alone, even when contrast venography is performed. In all cases in our report, a LA geometry of sufficient quality to guide ablation was successfully created with the FARAWAVE™ catheter, eliminating the need for contrast venography. As a result, atrial architecture was easily appreciable and anatomical variants were understood, namely the identification of common PV trunks and PV sub-branches. Without mapping, sub-branches or entire veins may be missed, leading to failure to apply PFA lesion sets to certain areas and incomplete PVI.

Compared to fluoroscopy alone, 3D-EAM allowed operators to appreciate the position of the FARAWAVE™ catheter within the LA geometry. Operators at times deemed adequate catheter positioning on fluoroscopy to, in fact, be associated with marked anterior or posterior tilt on 3D-EAM. By helping to visualise this in three-dimensional space, the catheter could be adjusted prior to PFA application, potentially improving the anatomical location of PFA delivery.

Prior to remapping with the FARAWAVE™ catheter, PV lesion tracking alone allowed for visual assessment of PV ablation coverage after initial PFA delivery, and the application of targeted touch-up lesions when gaps were identified. Through FARAWAVE™ voltage and ‘sparkle’ activation mapping, PV and ostial reconnections could be identified at procedure start, guiding the need for targeted repeat PV ablation, antral lesion set extension and/or the need for PWI.

Notably, in four cases, voltage maps created with the FARAWAVE™ and Advisor™ HD Grid catheters appeared concordant. This suggests that voltage mapping with the FARAWAVE™ catheter is feasible despite widely spaced bipolar electrodes and may be considered as an alternative to high-density mapping catheters for left atrial mapping, although further prospective validation is required. Indeed, due to the observed high fidelity in these initial cases, we subsequently used the FARAWAVE™ catheter solely for mapping in the remaining cases in our series. This was of particular utility in redo cases, where atrial substrate and PV reconnection could be assessed at procedure start, without the need for an additional high-density catheter.

Compared to traditional RF approaches, the relative ease and safety of performing PWI with the FARAWAVE™ catheter have led to the widespread uptake of this technique [15]. In our report, we performed PWI in six cases, ensuring complete PWI was achieved. During FARAPULSE™ PWI, care is required to ensure avoidance of the mitral annulus due to risk of coronary spasm [16]; 3D-EAM may facilitate this more effectively than fluoroscopic guidance, although further study is required. Due to the relatively large footprint of the FARAWAVE™ catheter, inadvertent encroachment of PFA applications onto the LA PW and/or roof at the time of PV isolation is possible. In some instances, this can result in partial—and unintended—PWI or roof lines, with gaps forming the substrate for atypical flutter circuits. This observation may explain the high rates of macro-reentrant atrial tachycardia circuits following FARAPULSE™ PVI [17].

### Potential limitations of mapping with the FARAWAVE™ catheter

Alongside procedural efficacy, healthcare efficiency is an important consideration during AF ablation. To determine the impact of 3D-EAM on procedural metrics, we compared procedural and fluoroscopy times to our previous experience of FARAPULSE™ PVI under fluoroscopic guidance [4].

Median skin-to-skin procedural time across all cases was 108 min, notably higher than in our prior experience of PFA under fluoroscopic guidance (68 min^4^). This was due to the additional time collecting a 3D-EAM prior to PFA applications. Interestingly, despite the integration with 3D-EAM, fluoroscopy times were similar to those from our previous experience without mapping (respectively, 20 min vs. 19 min^4^). Given the novelty and learning curve of 3D-EAM in this context, operators did not limit the use of fluoroscopy, often choosing to use this to confirm 3D-EAM findings and PFA catheter position. Indeed, as this 3D-EAM system does not recognise the deformation of the catheter in basket and flower configurations, operators were obliged to use fluoroscopy to determine the optimal catheter shape. For example, the system is unable to visualise FARAWAVE™ spline deformation when advancing into a PV, unlike on fluoroscopy. This is due to only electrode 3 of each spline being represented in the model, thereby rendering the catheter in a single-plane only within the 3D-EAM. Notably, when splines enter a PV, visual distortion of the splines within the EAM can occur due to a higher impedance inside the vein; as such, the catheter petals can sometimes appear of different sizes (Supplementary Material – Fig. 1C). This often resulted in the operator having to check the catheter position and direction on fluoroscopy. Finally, the system does also not allow for visualisation of the FARADRIVE™ sheath, requiring further fluoroscopic screening for optimal positioning. Future 3D-EAM iterations should seek to incorporate these features to facilitate reduced fluoroscopy times. Of note, the shortest fluoroscopy time in our series was 7 min, showing that low exposure times are achievable. Importantly, we did not utilise intracardiac echocardiography (ICE) in our series. ICE-guided PFA is an emerging technique [18], which, if combined with 3D-EAM in future practice, may enable reductions in—or elimination of—fluoroscopy. Indeed, the safety and efficacy of a zero-fluoro PFA procedure (using a combination of ICE and 3D-EAM) have recently been demonstrated in a retrospective study of 50 patients [19].

Cost—and cost-effectiveness more broadly—are crucial factors when considering adopting new technologies in the electrophysiology lab, particularly in publicly funded healthcare systems. We previously reported that, despite shorter procedural times with PFA compared to RF or cryoablation, PFA is considerably more expensive than thermal ablation, largely due to higher equipment cost (combined cost of the FARADRIVE™ sheath and FARAWAVE™ catheter is £4768) [4]. It is clear that 3D-EAM at the time of FARAPULSE™ AF ablation adds to this cost, through additional mapping equipment and systems. At our institution, the EnSite™ X EP System surface electrode kit costs £912. Our approach of mapping with the FARAWAVE™ catheter obviates the need to use expensive high-density mapping catheters (for example, the Advisor™ HD Grid catheter costing £2280). The additional £912 for the electrode kit in order to map with the FARAWAVE™ catheter *may* be justifiable based on the potential advantages we have described over fluoroscopic-only procedures, but must be demonstrated in prospective, cost-effectiveness studies taking into account the long-term efficacy of this approach.

### Study limitations

This was a single-centre, observational series, with small patient numbers. Indeed, our report aimed only to report the feasibility of mapping with the pentaspline PFA catheter itself. Further studies systemically assessing the utility of this approach in a large, prospective cohort are required before widespread adoption.

## Conclusion

Our report highlights the feasibility of 3D-EAM using the FARAWAVE™ catheter itself, discussing potential advantages and limitations of this approach over fluoroscopic-only guidance. This strategy may offer a number of solutions to important clinical issues during FARAPULSE™ AF ablation cases. Specifically, it may be useful in identifying conducting sleeves leading to incomplete PVI, guiding robust PWI, and lesion planning in redo cases. However, procedural duration was longer and fluoroscopy times were not improved, perhaps as a result of the learning curve associated with this approach. Further, additional cost may be a barrier to clinical implementation. These aspects may be addressed in the future with improved 3D-EAM iterations and workflow optimisation.

## Supplementary Information

Below is the link to the electronic supplementary material.

Supplementary file1 (DOCX 7923 KB)

Supplementary file2 (MP4 2523 KB)
