# Supplementary material for: An approach to electroanatomical mapping with a pentaspline pulsed field catheter to guide atrial fibrillation ablation
Source: J Interv Card Electrophysiol. 2025 Mar 4;68(4):921–31. doi: 10.1007/s10840-025-01980-6 (PMC12245958; doi:10.1007/s10840-025-01980-6)

**Supplementary Material for “Electroanatomical mapping with a pentaspline pulsed field catheter to guide atrial fibrillation ablation: Advantages and limitations”**

Mark T Mills^1,2^, Peter Calvert^1,2^, Calum Phenton^3^, Nicole Worthington^3^, Derick Todd^2^, Simon Modi^2^, Reza Ashrafi^2^, Richard Snowdon^2^, Dhiraj Gupta^1,2^, Vishal Luther^1,2^

^1^ Liverpool Centre for Cardiovascular Science at University of Liverpool, Liverpool John Moores University and Liverpool Heart & Chest Hospital, Liverpool, UK.

^2^ Department of Cardiology, Liverpool Heart & Chest Hospital NHS Foundation Trust, Thomas Drive, Liverpool L14 3PE, UK.

^3^ Abbott Medical UK Ltd, Solihull, UK.

**Address for Correspondence**

Dr Vishal Luther, Consultant Cardiologist & Electrophysiologist. Liverpool Centre for Cardiovascular Science, Liverpool Heart and Chest Hospital, Thomas Dr, Liverpool L14 3PE, UK. Email: [vishal.luther@lhch.nhs.uk](mailto:vishal.luther@lhch.nhs.uk)

Dr Mark Mills, Liverpool Centre for Cardiovascular Science at University of Liverpool, Liverpool John Moores University and Liverpool Heart & Chest Hospital, Liverpool, UK. Emails: [Mark.Mills@lhch.nhs.uk](mailto:Mark.Mills@lhch.nhs.uk) and [M.Mills@liverpool.ac.uk](mailto:M.Mills@liverpool.ac.uk)

**Index**

Page 2: **Supplementary Material – Table 1: Individual patient demographics and procedural characteristics.**

Page 3: **Supplementary Material – Figure 1: Visualisation of the FARAWAVE™ catheter in the EnSite™ EP system.**

Page 4: **Supplementary Material – Figure 2: Real-time catheter visualisation and shadowing.**

Page 5: **Supplementary Material – Figure 3**: **3D-EAM for posterior wall isolation.**

Page 6: **Supplementary Material – Video 1**: **Visualisation of guidewire tip in EnSite™ X.**

**Supplementary Material – Table 1: Individual patient demographics and procedural characteristics.**

| DEMOGRAPHICS | | | | | 3-DIMENSIONAL ELECTROANATOMICAL MAPPING | | | | NON-PULMONARY VEIN ABLATION | | PROCEDURAL METRICS | | | |
| --- | --- | --- | --- | --- | --- | --- | --- | --- | --- | --- | --- | --- | --- | --- |
| Patient | Age | AF type | LA dilatation | Re-do | HD grid pre | FW pre | HD grid post | FW post | PWI | CTI | Number of PFA deliveries | Skin-to-skin times (mins) | Fluoro time (mins) | Fluoro dose (cGy.cm^2^) |
| A | 63 | Paroxysmal | Normal |  | ✓ | ✓ | ✓ | ✓ |  |  | 40 | 130 | 22 | 921 |
| B | 44 | Persistent | Severe |  | ✓ | ✓ | ✓ | ✓ |  |  | 36 | 115 | 12 | 490 |
| C | 77 | Persistent | Severe |  | ✓ | ✓ | ✓ | ✓ | ✓ |  | 76 | 133 | 20 | 864 |
| D | 34 | Persistent | Mild | ✓ | ✓ | ✓ | ✓ | ✓ | ✓ |  | 61 | 182 | 26 | 1740 |
| E | 50 | Persistent | Severe |  | ✓ | ✓ |  | ✓ |  | ✓ | 52 | 110 | 32 | 1110 |
| F | 59 | Paroxysmal | Normal | ✓ |  | ✓ |  |  |  |  | 49 | 187* | 39 | 947 |
| G | 69 | Paroxysmal | Normal | ✓ |  | ✓ |  | ✓ |  |  | 38 | 104 | 20 | 258 |
| H | 61 | Paroxysmal | Mild | ✓ |  | ✓ |  |  |  |  | 43 | 94 | 28 | 1080 |
| I | 58 | Persistent | Severe | ✓ |  | ✓ |  | ✓ |  |  | 41 | 104 | 19 | 1040 |
| J | 74 | Paroxysmal | Normal | ✓ |  | ✓ |  | ✓ | ✓ |  | 36 | 90 | 12 | 398 |
| K | 71 | Paroxysmal | Normal |  |  | ✓ |  | ✓ |  |  | 40 | 93 | 7 | 309 |
| L | 58 | Persistent | Mild |  | ✓ |  |  | ✓ |  | ✓ | 45 | 106 | 8 | 220 |
| M | 73 | Paroxysmal | Mild |  |  | ✓ |  |  |  |  | 40 | 95 | 30 | 868 |
| N | 67 | Paroxysmal | Normal |  |  | ✓ |  | ✓ |  |  | 44 | 125 | 14 | 337 |
| O | 68 | Paroxysmal | Severe |  |  | ✓ |  | ✓ |  |  | 36 | 149 | 26 | 645 |
| P | 60 | Paroxysmal | Moderate |  |  | ✓ |  | ✓ |  | ✓ | 34 | 113 | 21 | 410 |
| Q | 58 | Paroxysmal | Mild |  |  | ✓ |  | ✓ |  |  | 38 | 95 | 12 | 256 |
| R | 32 | Paroxysmal | Normal |  |  | ✓ |  | ✓ |  |  | 42 | 91 | 16 | 1100 |
| S | 68 | Persistent | Severe |  |  | ✓ |  |  |  |  | 60 | 113 | 41 | 823 |
| T | 78 | Persistent | Moderate |  |  | ✓ |  | ✓ | ✓ |  | 52 | 91 | 12 | 282 |
| U | 68 | Persistent | Moderate |  |  | ✓ |  | ✓ | ✓ |  | 74 | 111 | 18 | 661 |
| V | 76 | Persistent | Moderate |  |  | ✓ |  | ✓ | ✓ |  | 64 | 82 | 11 | 273 |

*NB: Long procedure as electrophysiology study performed prior to decision for redo pulmonary vein isolation.

AF, atrial fibrillation; CTI, cavotricuspid isthmus; FW, FARAWAVE™ catheter; HD grid, Advisor™ HD grid catheter; LA, left atrium; PFA, pulsed field ablation; PWI, posterior wall isolation

**Supplementary Material – Figure 1: Visualisation of the FARAWAVE™ catheter in the EnSite™ EP system.** A: The FARAWAVE™ catheter is comprised of five splines, with four electrodes per spline. A bipole was derived for each of the five splines, with the distal electrode represented by the interpolation of electrodes 1, 2 and 4 of each spline, and electrode 3 as the proximal electrode. Electrode 3 on each spline is circled in blue. B: Electrode 3 of each spline visualised in the EnSite™ system (splines numbered from 1 to 5 on right-hand images). C: FARAWAVE™ catheter in EnSite™ X in the flower configuration, with impedance distortion affecting the visualisation of the splines.

**
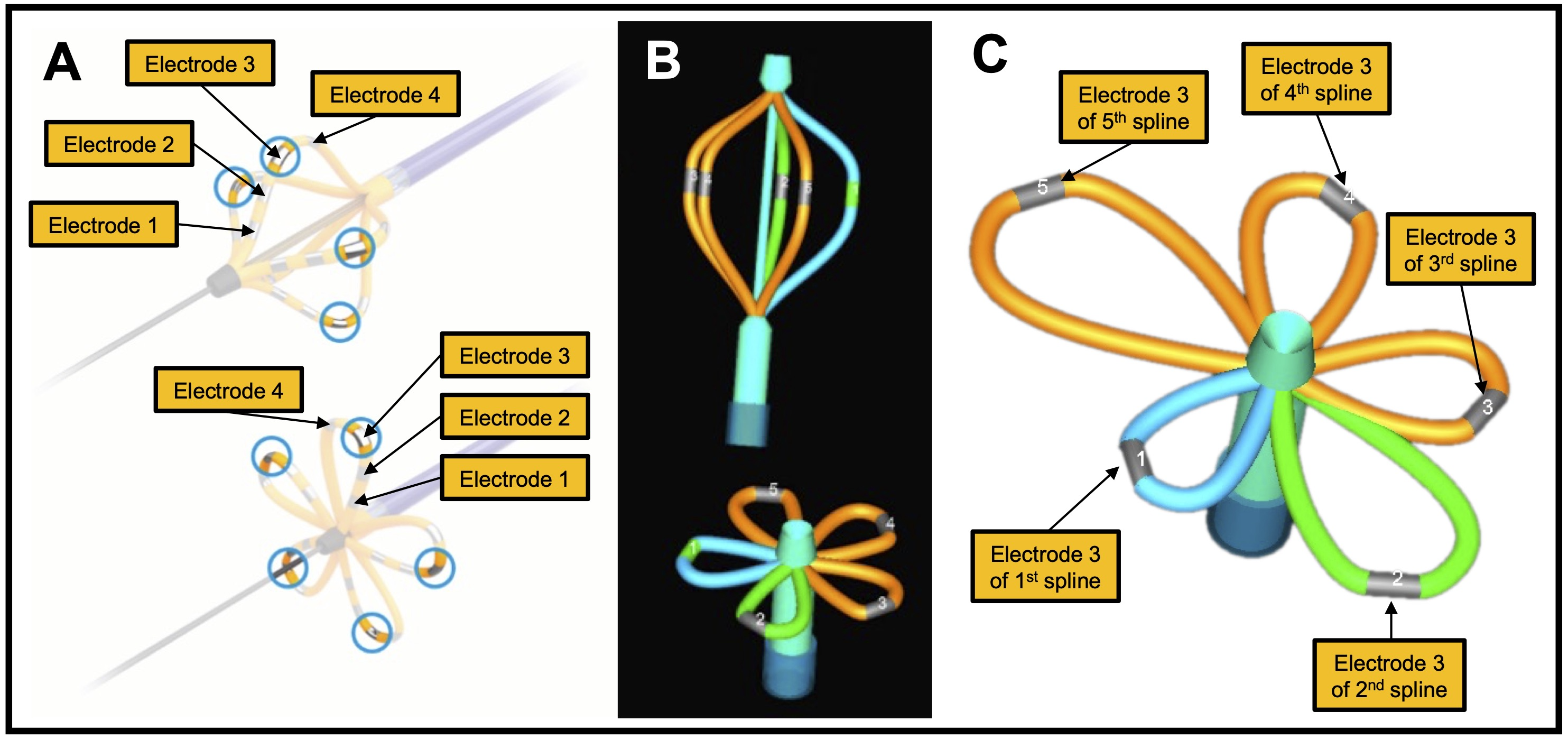
**

**Supplementary Material – Figure 2: Real-time catheter visualisation and shadowing.**  Visualisation of the FARAWAVE™ catheter in flower (top) and basket (lower) configurations within a FARAWAVE™-derived 3-dimensional electroanatomical map. Initial catheter position (left-hand images) can be ‘shadowed’, enabling visualisation of adequate rotation between applications (right-hand images).


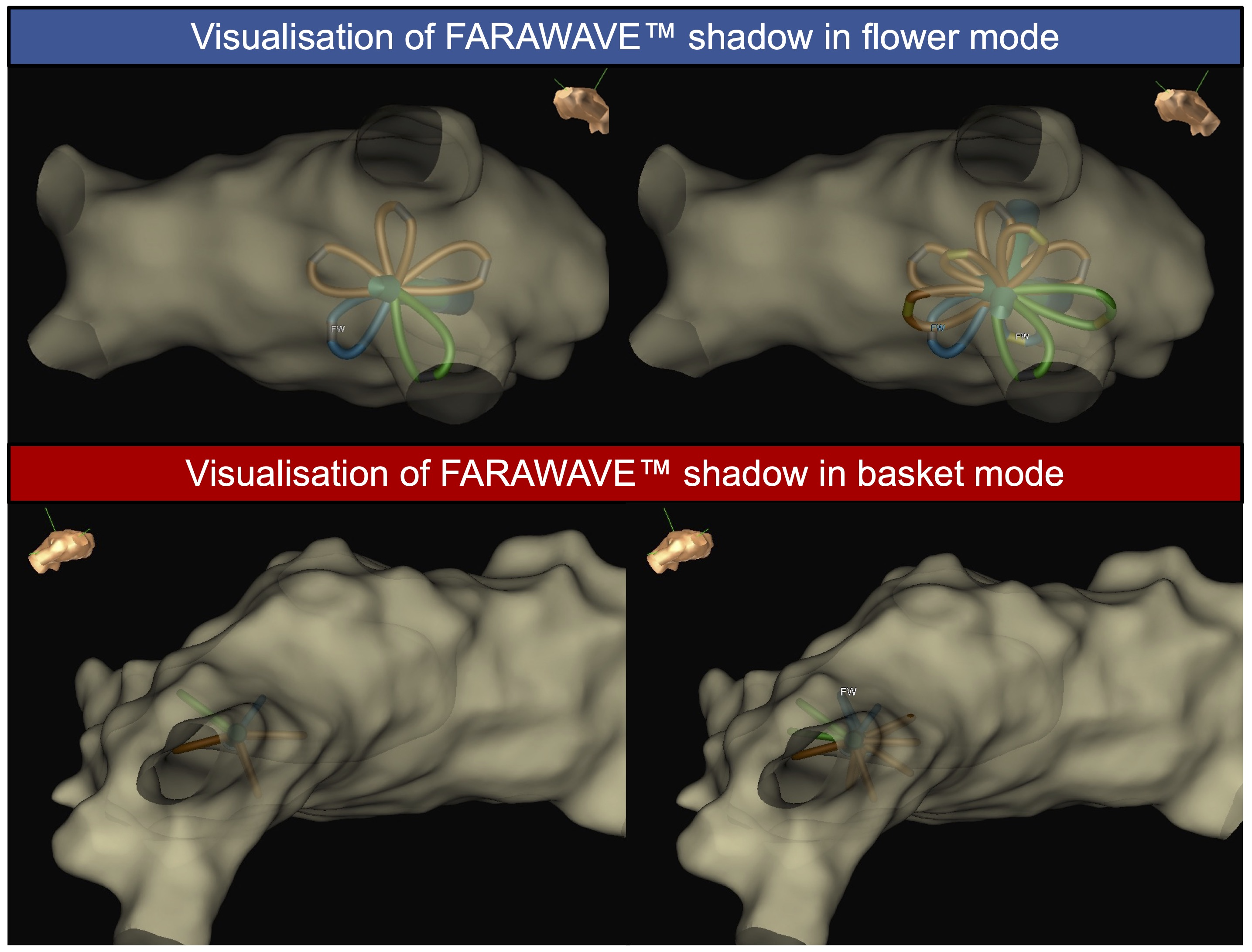


**Supplementary Material – Figure 3**: **3D-EAM for posterior wall isolation.** Postero-anterior views of the left atrium. Top left: FARAWAVE™ shadow map of catheter positions on the posterior wall and within the pulmonary veins. Top right: FARAWAVE™ lesion tracking map demonstrating good lesion coverage across the posterior wall. Bottom: FARAWAVE™ voltage map post-ablation demonstrating pulmonary vein and posterior wall isolation.


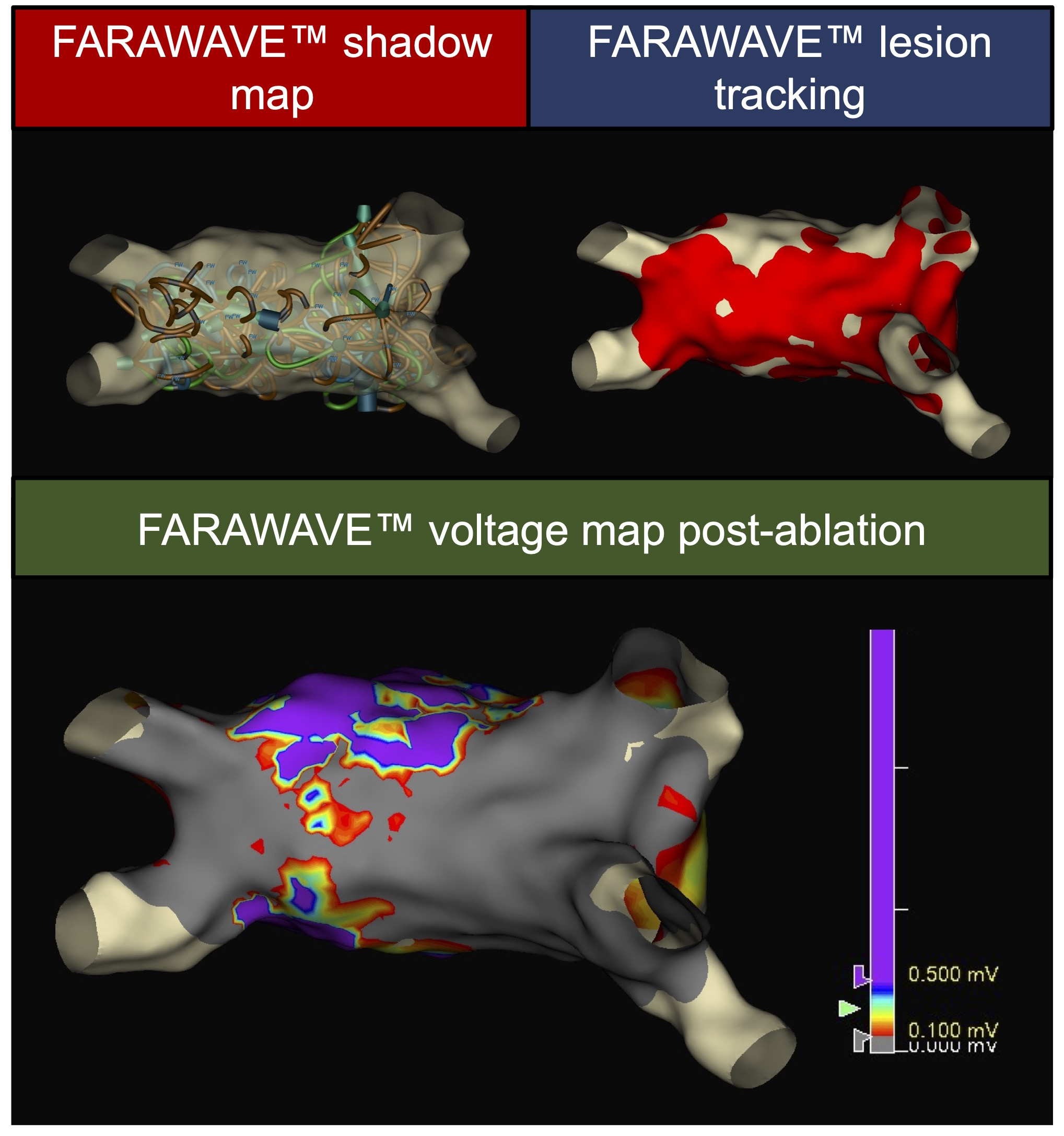


**Supplementary Material – Video 1**: **Visualisation of guidewire tip in EnSite™ X.** Illustration of guidewire tip (green circle) moving from lower branch of left upper pulmonary vein (LUPV) into upper branch of LUPV.


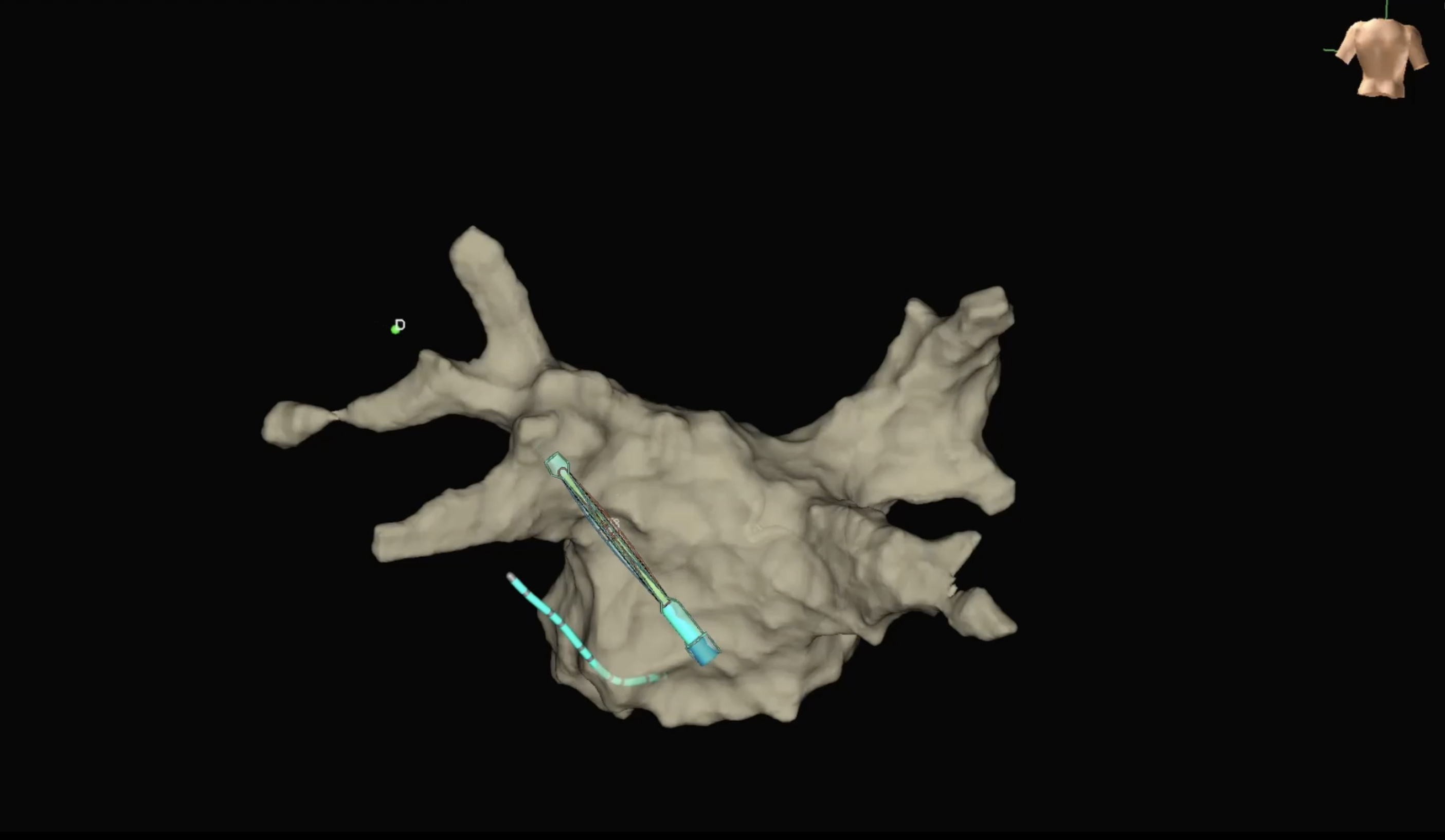

Supplement: Supplementary file 1 — Supplementary file1 (DOCX 7923 KB) [file 10840_2025_1980_MOESM1_ESM.docx]
